# Genetic Variants in Toll-Like Receptors Are Not Associated with Rheumatoid Arthritis Susceptibility or Anti-Tumour Necrosis Factor Treatment Outcome

**DOI:** 10.1371/journal.pone.0014326

**Published:** 2010-12-15

**Authors:** Marieke J. H. Coenen, Christian Enevold, Pilar Barrera, Mascha M. V. A. P. Schijvenaars, Erik J. M. Toonen, Hans Scheffer, Leonid Padyukov, Alf Kastbom, Lars Klareskog, Anne Barton, Wietske Kievit, Maarten J. Rood, Tim L. Jansen, Dorine Swinkels, Piet L. C. M. van Riel, Barbara Franke, Klaus Bendtzen, Timothy R. D. J. Radstake

**Affiliations:** 1 Department of Human Genetics, Radboud University Nijmegen Medical Centre, Institute for Genetic and Metabolic Disease (IGMD), Nijmegen, The Netherlands; 2 Department of Rheumatology, Institute for Inflammation Research, Rigshospitalet, University Hospital of Copenhagen, Copenhagen, Denmark; 3 Department of Rheumatology, Radboud University Nijmegen Medical Centre, Nijmegen, The Netherlands; 4 Rheumatology Unit, Department of Medicine, Karolinska Institutet at Karolinska University Hospital Solna, Stockholm, Sweden; 5 Division of Rheumatology, Department of Clinical and Experimental Medicine, Linköping University, Linköping, Sweden; 6 Arthritis Research Campaign Epidemiology Unit, The University of Manchester, Manchester, United Kingdom; 7 Department of Rheumatology, Ziekenhuis Gelderse Vallei, Ede, The Netherlands; 8 Department of Rheumatology, Medical Centre Leeuwarden, Leeuwarden, The Netherlands; 9 Laboratory of Clinical Chemistry, Laboratory Medicine, Radboud University Nijmegen Medical Centre, Nijmegen, The Netherlands; University of Medicine and Dentistry of New Jersey, United States of America

## Abstract

**Background:**

Several studies point to a role of Toll-like receptors (TLRs) in the development of rheumatoid arthritis (RA). We investigated if genetic variants in *TLR* genes are associated with RA and response to tumour necrosis factor blocking (anti-TNF) medication.

**Methodology and Principal Findings:**

22 single nucleotide polymorphisms (SNPs) in seven *TLR* genes were genotyped in a Dutch cohort consisting of 378 RA patients and 294 controls. Significantly associated variants were investigated in replication cohorts from The Netherlands, United Kingdom and Sweden (2877 RA patients and 2025 controls). 182 of the Dutch patients were treated with anti-TNF medication. Using these patients and a replication cohort (269 Swedish patients) we analysed if genetic variants in *TLR* genes were associated with anti-TNF outcome. In the discovery phase of the study we found a significant association of SNPs rs2072493 in *TLR5* and rs3853839 in *TLR7* with RA disease susceptibility. Meta-analysis of discovery and replication cohorts did not confirm these findings. SNP rs2072493 in *TLR5* was associated with anti-TNF outcome in the Dutch but not in the Swedish population.

**Conclusion:**

We conclude that genetic variants in *TLRs* do not play a major role in susceptibility for developing RA nor in anti-TNF treatment outcome in a Caucasian population.

## Introduction

Rheumatoid arthritis (RA) is a severe chronic inflammatory disorder leading to joint damage. The causes of RA are largely unknown, however, the role of genetic factors is evident, with the MHC region as the major contributor [1]. To date, more than 20 non-MHC regions explaining approximately one third of the genetic contribution to RA have been identified [2].

The role of Toll-like receptors (TLRs) in the development of RA is supported by several studies. Synovial fibroblasts of RA patients constitutively express TLR 1-6, and it has been demonstrated that TLR 2, 3, 4 and 7 are up-regulated in RA synovial tissue compared to that of osteoarthritis patients and healthy controls [3]. Furthermore, the ligands for TLRs have been detected in synovium of RA patients [4],[5]. Since TLRs are potent activators of pro-inflammatory cytokines, including tumour necrosis factor (TNF) alpha, this also makes them interesting candidates for treatment outcome prediction, especially for TNF-neutralizing therapy [6].

Several groups explored the contribution of genetic variants in *TLR* genes to the development of RA. A Korean study showed an association of a dinucleotide repeat in *TLR2* with RA [7]. One other study focusing on two SNPs in *TLR2* resulting in amino acid substitutions (Arg677Trp and Arg753Gln) could not detect an association between these rare variants and RA [8]. Although conflicting results have been reported on the functional variant Asp299Gly (rs4986790) in *TLR4* most studies showed no association of this variant with RA susceptibility [8]–[13]. A French group investigated 10 SNPs in *TLR1*, *2*, *4*, *6* and *9* in 100 families but did not find evidence for an association of variants in these genes with RA, autoantibody production or erosions [13]. A Turkish study, investigating variants in TLR3, 9 and 10 in 100 patients, showed an association between a variant in TLR9 and RA susceptibility [14]. So far, only one small pharmacogenetic study on *TLRs* has been published [15]. The authors did not demonstrate an association between the Asp299Gly variant in *TLR4* and the response to disease modifying anti-rheumatic drugs (DMARDs).

Although most studies performed to date point to a lack of association between genetic variants in genes coding for *TLRs* and RA disease susceptibility or treatment response, these have either been very small or did not comprehensively test potentially functional SNPs in the genes. Therefore we performed an association study of *TLRs* testing involvement in RA pathogenesis and anti-TNF treatment response including seven *TLR* genes, three of which (*TLR5*, *7* and *8*) have not been investigated in connection with RA before. The twenty-two SNPs investigated were selected for potential effects on gene function or regulation.

## Results

### Case control analysis

In the discovery phase of our study we included 378 RA patients and 294 controls and genotyped 22 SNPs in seven *TLR* genes. Genotyping failed for 51 samples (22 cases and 26 controls). Six SNPs were excluded from the analysis, based on a low MAF (Table 1).

**Table 1:** Results of the case control association analysis in the Dutch discovery set.

gene	chromosome	SNP	BP	functional effect	major allele	minor allele	MAF cases	MAF controls	chi-square	p-value	OR	95% CI interval
TLR2	4	rs1898830	154827903	Promoter (−15607)	A	G	0.34	0.33	0.05	0.83	1.03	0.81–1.30
		rs5743704	154845401	Pro631His	C	A	0.04	0.05	n.a.	n.a.	n.a.	n.a.
		rs5743708	154845767	Arg753Gln	G	A	0.04	0.04	n.a.	n.a.	n.a.	n.a.
TLR3	4	rs3775291	187241068	Leu412Phe	C	T	0.30	0.30	0.01	0.92	1.01	0.79–1.29
TLR4	9	rs4986790	119515123	Asp299Gly	A	G	0.05	0.05	0.003	0.96	0.99	0.60–1.63
		rs4986791	119515423	Thr399Ile	C	T	0.05	0.05	0.003	0.96	0.99	0.60–1.63
		rs7873784	119518757	3′ UTR	G	C	0.18	0.16	0.54	0.46	1.12	0.83–1.51
TLR5	1	rs5744176	221350916	Asp694Gly	A	G	0	0	n.a.	n.a.	n.a.	n.a.
		rs5744174	221351151	Phe616Leu	T	C	0.47	0.46	0.12	0.73	1.04	0.83–1.30
		**rs2072493**	**221351222**	**Asn592Ser**	**A**	**G**	**0.10**	**0.15**	**6.35**	**0.01**	**0.64**	**0.46–0.91**
		rs5744168	221351823	Arg392STOP	C	T	0.08	0.07	0.74	0.39	1.21	0.78–1.88
		rs764535	221352752	Thr82Ile	G	A	0.02	0.01	n.a.	n.a.	n.a.	n.a.
TLR7	X	rs2302267	12795499	Exon/intron boundary	T	G	0.05	0.04	n.a.	n.a.	n.a.	n.a.
		rs179008	12813580	Gln11Leu	A	T	0.21	0.24	0.94	0.33	0.86	0.63–1.17
		rs5743781	12814891	Ala448Val	C	T	0	0.0003	n.a.	n.a.	n.a.	n.a.
		**rs3853839**	**12817579**	**3′ UTR**	**C**	**G**	**0.13**	**0.21**	**8.95**	**0.003**	**0.59**	**0.42–0.84**
TLR8	X	rs5741883	12834142	Promoter (−605)	C	T	0.24	0.22	0.69	0.41	1.14	0.84–1.55
		rs3764879	12834618	Promoter (−129)	C	G	0.22	0.26	2.07	0.15	0.80	0.59–1.08
		rs3764880	12834747	Exon (−3679)	A	G	0.22	0.26	1.87	0.17	0.81	0.60–1.10
		rs5744088	12850485	3′ UTR	G	C	0.16	0.13	1.49	0.22	1.26	0.87–1.82
TLR9	3	rs5743836	52235822	Promoter (−1237)	A	G	0.16	0.18	1.21	0.27	0.85	0.63–1.14
		rs187084	52236071	Promoter (−1486)	A	G	0.41	0.43	0.49	0.48	0.92	0.73–1.16

MAF: minor allele frequency.

OR: odds ratio.

CI: confidence interval.

n.a.: not analysed.

**Figure 1:**
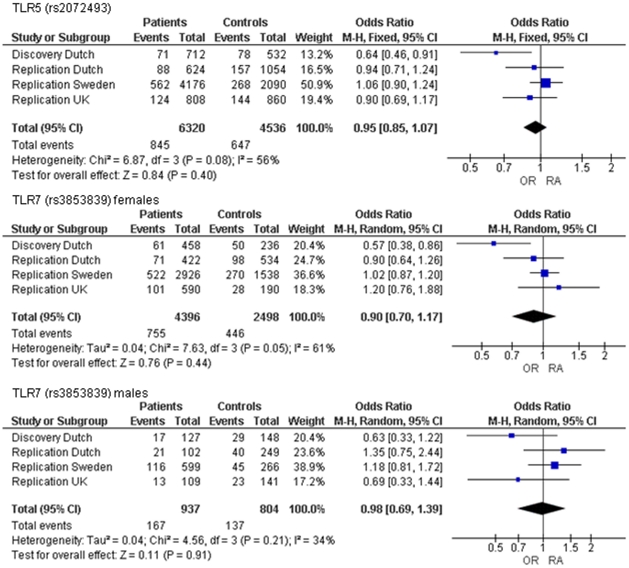
Meta-analysis of case control studies for TLR5 rs2072493 and TLR7 rs3853839.

Two of the investigated polymorphisms, rs2072493 in *TLR5* and rs3853839 in *TLR7*, showed nominally significant allelic association with RA (p≤0.01; Table 1). The minor allele of both SNPs conferred a protective effect for the development of RA. A stratified analysis for either the shared epitope or rheumatoid arthritis. We did not identify SNPs that were specifically associated with one of the RA subgroups. The odds ratios for SNPs rs2072493 and rs3853839 were comparable to the odds ratios observed in the complete patient population (data not shown). The frequencies of rs2072493 and rs3853839 were investigated in three additional cohorts from The Netherlands, Sweden and the UK, consisting of 2877 patients and 2025 controls. This analysis showed no association of the SNPs in *TLR5* and *TLR7* with RA aetiology either in the separate cohorts or in a meta-analysis (Figure 1).

### Anti-TNF treatment response

A subsample of the Dutch patients were treated with anti-TNF medication and had EULAR response information available at 3 months (n = 182, Table 2). Results of the association analysis are shown in Table 3. *TLR5* rs2072493 showed a significant association with anti-TNF treatment outcome (p = 0.003). In total, 182 patients were included in the analysis. The EULAR response criteria were used to asses association of a genotype with anti-TNF response. The test statistic and p-value were from Fisher's Exact Test for most SNPs, those indicated with * were based on Pearson Chi-square and p-value. A medication specific analysis showed that none of the investigated SNPs was associated with a specific anti-TNF (Table S1). Validation of association found with TLR5 in 269 RA patients treated with anti-TNF from Sweden did not confirm our initial findings (Fisher's exact test p = 0.63).

**Table 2:** Patient characteristics of RA patients treated with anti-TNF medication.

Cohort	The Netherlands	Sweden
Number of patients (N (%))	182 (100)	269 (100)
Gender (female)	122 (67.0)	211 (78.4)
RF factor positive	141 (77.5)#	169 (88.9)##
MTX co-medication	105 (61.0)###	185 (68.8)
Anti-TNF medication		
Adalimumab	61 (33.5)	0 (0)
Infliximab	118 (64.8)	156 (58.0)
Etanercept	3 (1.6)	113 (42.0)
Good response*	45 (24.7)	76 (28.3)
Moderate response*	92 (50.5)	137 (50.9)
No response*	45 (24.7)	56 (20.8)
DAS28 baseline (mean ± SD)	5.72±1.16	5.84±1.13
DAS28 3 months after treatment initiation (mean ± SD)	4.12±1.36	3.89±1.30

*Response based on EULAR response criteria.

Data available for ^#^180, ^##^190 and ^###^172 patients.

**Table 3:** Results of the association analysis for anti-TNF treatment outcome after three months of treatment initiation.

Gene	chromosome	SNP	Gender	Test statistic	p-value
TLR2	4	rs1898830		11.188*	0.024*
		rs5743704		n.a.	n.a.
		rs5743708		n.a.	n.a.
TLR3	4	rs3775291		7.74	0.096
TLR4	9	rs4986790		5.32	0.19
		rs4986791		5.32	0.19
		rs7873784		1.28	0.886
TLR5	1	rs5744176		n.a.	n.a.
		rs5744174		5.77	0.22
		rs2072493		**13.52**	**0.003**
		rs5744168		2.15	0.72
		rs764535		n.a.	n.a.
TLR7	X	rs2302267		n.a.	n.a.
		rs179008	males	not polymorph	not polymorph
			females	2.97*	0.24*
		rs5743781		n.a.	n.a.
		rs3853839	males	4.69	0.089
			females	4.61	0.27
TLR8	X	rs5741883	males	0.87	0.75
			females	3.34	0.51
		rs3764879	males	1.41	0.48
			females	1.76	0.85
		rs3764880	males	1.41	0.48
			females	1.76	0.85
		rs5744088	males	0.63	0.80
			females	3.20	0.50
TLR9	3	rs5743836		2.98	0.63
		rs187084		0.61	0.97

In total, 182 patients were included in the analysis. The EULAR response criteria were used to asses association of a genotype with anti-TNF response. The test statistic and p-value were from Fisher's Exact Test for most SNPs, those indicated with * were based on Pearson Chi-square and p-value. SNPs located on the X-chromosome were analysed for males and females separately.

The differences observed between the Dutch and Swedish cohort were not related to the anti-TNF used (Fisher's exact test Swedish cohort for infliximab (p = 0.75) and etanercept (p = 0.49)).

## Discussion

We found no evidence for association between 22 SNPs in *TLR* genes and RA disease susceptibility or anti-TNF treatment response, in keeping with the majority of the other candidate gene studies investigating *TLRs* in RA patients [7]–[13]. Furthermore, genome-wide association studies (GWAS) do not report associations between *TLR* genes and RA disease susceptibility (www.genome.gov/gwastudies). Accessed December 31^th^ 2009), suggesting that genetic variants in these genes are not a major contributor to disease development [16]. However, some TLR genes were poorly covered in the initial GWAS (e.g. no SNPs in TLR5 and 9 were included in these studies) [17],[18]. The *TLR* genes remain strong candidates for susceptibility genes in RA given their crucial role in mediating inflammatory signalling. One possibility is that these genes might harbour rare variants with large effect sizes, which would not be well tagged with the common SNPs selected for investigation in the current study. With the introduction of next generation sequencing technologies it should become possible to analyse the genes in a thorough manner but much larger sample sizes will be required to ensure power to detect association with rare variants, even if effect sizes are larger.

We could not identify an association of the investigated SNPs with anti-TNF response. We were unable to replicate the initial finding in the Dutch cohort. Differences in ethnicity, gender, RF and co-medication might influence the observed discrepancies. Besides the study has low power to detect an association due to the small patient population. Despite this the function of *TLRs* makes them likely candidate genes, since they directly modulate TNF expression [6]. In addition, De Rijcke and colleagues showed that anti-TNF treatment in spondylarthropathy results in a reduced expression of *TLR2* and *TLR4* [19]. The TLR pathway should therefore be investigated in more detail before drawing definite conclusions about the contribution of these genes to anti-TNF treatment outcome.

In conclusion, our study does not support an association between genetic variants in *TLR* genes and RA disease susceptibility or anti-TNF treatment response.

## Materials and Methods

### Ethics Statement

The study was approved by the ethics committees of the participating institutions. For the Dutch samples the “Commissie Mensgebonden Onderzoek (CMO) Regio Arnhem Nijmegen” of the Radboud University Nijmegen Medical Centre approved the study (CMO number 2004/014). All Dutch patients and controls gave written informed consent prior to participation in the study. Verbal consent was received from all patients participating in the EIRA study and it was registered at clinical journals according to Swedish law. Written consent was received from healthy control individuals. The ethics committee of Karolinska Institutet approved the study. All UK patients and controls were recruited with ethical approval and provided written informed consent (North-West Multi-Centre Research Ethics Committee (MREC 99/8/84) and the University of Manchester Committee on the Ethics of Research on Human Beings).

### Patients and controls

All patients in this study fulfilled the American College of Rheumatology (ACR) criteria. In the discovery phase 378 Dutch RA patient participating in the Nijmegen Inception cohort and 294 anonymous blood donors were included [20]. Cohorts from The Netherlands, Sweden and England were included in the replication phase. The Dutch replication cohort consisted of 313 RA patients participating in the Dutch Rheumatoid Arthritis Monitoring (DREAM) registry [21]. The Dutch controls (n = 527) were part of the Nijmegen Biomedical Study [22].

The Swedish patients (n = 2158) and controls (sex, age and residence area matched, n = 1068) were part of the Epidemiological Investigation of Rheumatoid arthritis (EIRA) study. The UK patients (n = 406) were recruited as part of the Arthritis Research Campaign National Repository of patients and families with one patient from each family selected for genotyping. UK controls without a history of inflammatory arthritis (n = 430) were recruited from General Practitioner records or from blood donors.

### Anti-TNF treatment outcome

Patients participating in the DREAM registry were included in the study of anti-TNF treatment response. The registry includes patients with RA who start with anti-TNF treatment, according to the Dutch recommendations (Disease Activity Score (DAS28) >3.2 and previous failure on at least two disease-modifying antirheumatic drugs (DMARDs), one of which has to be methotrexate (MTX)) [21]. The DAS28 at treatment start and after 3 months of treatment were used to calculate the clinical response according to the EULAR criteria [23]. Association analysis was performed for the total patient group and a subgroup analysis for the patients treated with infliximab and adalimumab. Nominally significant findings (p≤0.01) were validated in 269 anti-TNF treated RA patients from Sweden [23].

### SNP genotyping

Twenty-two SNPs located in *TLR2*, *3*, *4*, *5*, *7*, *8* and *9* were genotyped in all Dutch patients and Dutch anonymous blood donors (Table 1). SNP selection criteria are described previously [24]. SNPs were genotyped using two multiplexed bead-based assays on a Luminex 100IS flow cytometer (Luminex Corporation, Austin, TX, USA). The tests were based on procedures previously described, with some modifications [24]–[26].

Replication studies were performed using TaqMan® SNP genotyping assays for SNPs rs2072493 in *TLR5* (assay ID C__22273027_10) and rs3853839 in *TLR7* (assay ID C___2259573_10).

### Statistical analysis

For quality control reasons we excluded SNPs that had a minor allele frequency (MAF) <0.05. Hardy-Weinberg Equilibrium (HWE) was tested using the controls (only females were used for X-chromosomal SNPs), no deviations were observed. The whole genome association analysis toolset (PLINK) was used for association analysis at the allelic level [27]. A stratified analysis was performed for RF positive and negative as well as shared epitope positive and negative patients. SNPs with a nominal p-value ≤0.01 in the discovery sample were included in the replication phase. Review Manager 5 (Review Manager (RevMan) Version 5.0. Copenhagen: The Nordic Cochrane Centre, The Cochrane Collaboration, 2008) was used to perform a meta-analysis. Homogeneity of odds ratios (OR) among cohorts was calculated using the Breslow-Day method, and pooled ORs were calculated under a fixed effects model (Mantel-Haenszel meta-analysis).

To assess the effect of SNPs on anti-TNF response, a two-tailed Fisher's exact or Chi-square test was used (where applicable) for the comparison of the EULAR response scores between the three genotype groups in a 3×3 table in SPSS (SPSS for Windows, version 16.0). X-chromosomal SNPs were analyzed for males and females separately.

## Supporting Information

Table S1 Results of the association analysis for anti-TNF treatment outcome after three months of treatment initiation.
(0.07 MB DOC)
