# Supplementary material for: Genetic Variants in Toll-Like Receptors Are Not Associated with Rheumatoid Arthritis Susceptibility or Anti-Tumour Necrosis Factor Treatment Outcome
Source: PLoS One. 2010 Dec 15;5(12):e14326. doi: 10.1371/journal.pone.0014326 (PMC3002281; doi:10.1371/journal.pone.0014326)
Supplement: Table S1 — Results of the association analysis for anti-TNF treatment outcome after three months of treatment initiation. (0.07 MB DOC) [file pone.0014326.s001.doc]

Results of the association analysis for anti-TNF treatment outcome after three months of treatment initiation.

| Gene | chr | SNP | Gender | Test statistic (p-value)  (all patients (n=182)) | Test statistic (p-value)  (infliximab (n=118)) | Test statistic (p-value)  (adalimumab (n=61)) |
| --- | --- | --- | --- | --- | --- | --- |
| TLR2 | 4 | rs1898830 |  | 11.188 (0.024)* | 7.86 (0.097)* | 6.2 (0.17) |
|  |  | rs5743704 |  | n.a. | n.a. | n.a. |
|  |  | rs5743708 |  | n.a. | n.a. | n.a. |
| TLR3 | 4 | rs3775291 |  | 7.74 (0.096) | 5.90 (0.20) | 7.53 (0.087) |
| TLR4 | 9 | rs4986790 |  | 5.32 (0.19) | 2.85 (0.66) | 4.35 (0.35) |
|  |  | rs4986791 |  | 5.32 (0.19) | 2.85 (0.66) | 4.35 (0.35) |
|  |  | rs7873784 |  | 1.28 (0.886) | 3.19 (0.53) | 3.10 (0.62) |
| TLR5 | 1 | rs5744176 |  | n.a. | n.a. | n.a. |
|  |  | rs5744174 |  | 5.77 (0.22) | 8.09 (0.83) | 1.89 (0.77) |
|  |  | rs2072493 |  | **13.52 (0.003)** | **8.80 (0.031)** | 7.55 (0.064) |
|  |  | rs5744168 |  | 2.15 (0.72) | 2.25 (0.73) | 1.58 (0.51) |
|  |  | rs764535 |  | n.a. | n.a. | n.a. |
| TLR7 | X | rs2302267 |  | n.a. | n.a. | n.a. |
|  |  | rs179008 | males | not polymorph | not polymorph | not polymorph |
|  |  |  | females | 2.97 (0.24)* | 1.34 (0.52) | 2.44 (0.32) |
|  |  | rs5743781 |  | n.a. | n.a. | n.a. |
|  |  | rs3853839 | males | 4.69 (0.089) | 3.07 (0.25) | 1.87 (0.50) |
|  |  |  | females | 4.61 (0.27) | 0.60(0.81) | 5.30 (0.23) |
| TLR8 | X | rs5741883 | males | 0.87 (0.75) | 1.33 (0.59) | 1.21 (0.74) |
|  |  |  | females | 3.34 (0.51) | 3.79 (0.44) | 3.62 (0.49) |
|  |  | rs3764879 | males | 1.41 (0.48) | 1.32 (0.48) | 0.43 (1.0) |
|  |  |  | females | 1.76 (0.85) | 0.77 (0.66)* | 4.76 (0.27) |
|  |  | rs3764880 | males | 1.41 (0.48) | 1.32 (0.48) | 0.43 (1.0) |
|  |  |  | females | 1.76 (0.85) | 0.77 (0.68)* | 4.76 (0.27) |
|  |  | rs5744088 | males | 0.63 (0.80) | 1.29 (0.56) | 0.88 (1.0) |
|  |  |  | females | 3.20 (0.50) | 2.57 (0.67) | 2.44 (0.33) |
| TLR9 | 3 | rs5743836 |  | 2.98 (0.63) | 2.83 (0.67) | 0.32 (1.0) |
|  |  | rs187084 |  | 0.56 (0.96)* | 0.42 (0.98)* | 1.10 (0.92) |

The EULAR response criteria were used to asses association of a genotype with anti-TNF response. The test statistic and p-value were from Fisher’s Exact Test for most SNPs, those indicated with * were based on Pearson Chi-square and p-value. SNPs located on the X-chromosome were analysed for males and females separately. Chr: chromosome
